# ﻿*Geosesarma wongi* sp. nov., a distinctive new species of semiterrestrial crab from Tapah Hills in Perak, Peninsular Malaysia; with notes on *G. peraccae* (Nobili, 1903) and *G. cataracta* Ng, 1986 (Crustacea, Brachyura, Sesarmidae)

**DOI:** 10.3897/zookeys.1266.172575

**Published:** 2026-01-14

**Authors:** Peter K. L. Ng

**Affiliations:** 1 Lee Kong Chian Natural History Museum (LKCNHM), 2 Conservatory Drive, Singapore 117377, Singapore Lee Kong Chian Natural History Museum (LKCNHM) Singapore Singapore

**Keywords:** Comparative taxonomy, freshwater crab, Malay Peninsula, new taxon, sesarmid crab, Southeast Asia, taxonomy

## Abstract

A new species of semiterrestrial freshwater crab is described from Tapah Hills in Peninsular Malaysia. *Geosesarma wongi***sp. nov.** is the 13^th^ member of the genus reported from the country and is most similar to *G. peraccae* (Nobili, 1903) and *G. cataracta* Ng, 1986, but easily separated by its distinct carapace features and structures of the adult male chela and male first gonopod. The types of *G. peraccae* are figured for the first time. *Geosesarma cataracta* is also figured in detail, and the type series is shown to be mixed.

## ﻿Introduction

Twelve species of the semiterrestrial sesarmid genus *Geosesarma* De Man, 1892, are known from Peninsular Malaysia: *G. albomita* Yeo & Ng, 1999 (Pulau Tioman), *G. bunian* Ng, Khadijah-Ahmad & Ahmad, 2025 (Kedah), *G. cataracta* Ng, 1986, *G. faustum* Ng, 2017 (Penang), *G. foxi* (Kemp, 1918) (Langkawi), *G. johnsoni* (Serène, 1968) (Penang), *G. malayanum* Ng & Lim, in Ng, 1986 (Johor, Selangor, Pahang, Perak, Terengganu), *G. penangense* (Tweedie, 1940) (Penang), *G. peraccae* (Nobili, 1903) (Johor, Pahang, Singapore), *G. serenei* Ng, 1986 (Perak), *G. scandens* Ng, 1986 (Pahang), and *G. tiomanicum* Ng, 1986 (Pulau Tioman) (cf. Ng 1988; Yeo and Ng 1999; Ng 2004, 2017; Ng et al. 2025). One more species described from swamp forests in southernmost Thailand, *G. tondaeng* Ng, Yeesin & Promdam, 2023, is likely to also occur in Peninsular Malaysia, although it has not been formally recorded.

A new species is here described from Tapah Hills in Perak. *Geosesarma wongi* sp. nov. has a distinctive coloration (adult males have a dark carapace with bright orange chelipeds), as well as a diagnostic male chela and first gonopods. The new species appears to be closest morphologically to *G. cataracta* Ng, 1986, from Bukit Larut in Perak but can easily be distinguished by various morphological characters. The description of the new species forms the basis of the present paper.

## ﻿Material and methods

Specimens examined are deposited in the Zoological Reference Collection (**ZRC**) of the Lee Kong Chian Natural History Museum, National University of Singapore; and the Museo Regionale di Scienze Naturali (**MRSN**), Torino, Italy. The terminology used follows Ng (1988) and Davie et al. (2015). Measurements, in millimetres, are of the maximum carapace width and length, respectively. The following abbreviations are used – asl: above sea level; coll.: collected by; G1: male first gonopod: G2: male second gonopod: P2–P5: pereopods 2–4 (= ambulatory legs 1–4), respectively.

## ﻿Taxonomy

### ﻿Family Sesarmidae Dana, 1851

#### ﻿Genus Geosesarma

Taxon classificationAnimaliaDecapodaSesarmidae

De Man, 1892

F4EB0277-C076-5B7A-BBFF-5A7170710C9B

##### Type species.

Sesarma (Geosesarma) nodulifera De Man, 1892, subsequent designation by Serène and Soh (1970).

#### ﻿ Geosesarma wongi sp. nov.

Taxon classificationAnimaliaDecapodaSesarmidae

91E6CB1F-1BB5-5703-B18D-3EB3037717FC

https://zoobank.org/542BD346-FF5B-4F20-8D56-2FF934D8A160

Figs 1, 2, 3, 4, 5

##### Material examined.

**Holotype**: • male (12.7 × 11.3 mm) (ZRC 2025.0023), forest near 16^th^ milestone, Tapah Hill, east of Jor Main Dam, near Sungai Sekam, east of Highway 59 to Tapah, Batang Padang area, Perak, Peninsular Malaysia, 4.3491°N, 101.3481°E, ca. 550 m asl, coll. aboriginal collectors, July 2025. **Paratypes**: • 7 males, 11 females, 1 ovigerous female (12.6 × 11.0 mm, with 4 eggs, each ca. 1.45 mm) (ZRC 2025.0027), same data as holotype.

##### Comparative material.

*Geosesarma peraccae* (Nobili, 1903): **lectotype** • male (11.8 × 9.8 mm) (MRSN Cru 1421a), Singapore, coll. Deschamps; **paralectotype** • female (9.1 × 7.9 mm) (MRSN Cru 1421b), same data as lectotype; 1 male (11.8 × 10.4 mm), 1 female (12.6 × 10.7 mm) (ZRC 1990.0518), Nee Soon Swamp Forest, Singapore, coll. P.K.L. Ng, February 1990. *Geosesarma cataracta* Ng, 1986: **holotype** • male (12.4 × 10.9 mm) (ZRC 1985.1760), Bukit Larut (Maxwell Hill), near Taiping, Perak, 4°47'N, 100°45'E, ca. 350 m, under mats of bryophytes and algae on damp area adjacent a waterfall, coll. H.P. Ng & P.K.L. Ng, September 1984; **paratypes**: • 3 males, 4 females (largest 10.5 × 9.5 mm), 3 juveniles (ZRC 1985.1762–1771), same data as holotype.

##### Diagnosis.

Carapace rectangular, wider than long, width to length ratio 1.11–1.14, lateral margins almost straight, gently divergent towards posterior carapace margin (Fig. 2A, B); dorsal surfaces with well-defined regions, anterior half with prominent low, rounded tubercles and granules; postfrontal lobes distinct, median lobes with transverse row of relatively dense short setae, setae sparser on lateral lobes; epibranchial region raised; protogastric region gently convex; urogastric region raised, with gastrocardiac groove deep (Fig. 2A, B); frontal margin distinctly deflexed, frontal lobes broad, with gently convex margins in dorsal view, separated by wide shallow median concavity; postfrontal cristae sharp, distinct (Fig. 2A–C); external orbital angle triangular, directed anteriorly, not extending beyond lateral carapace margin, outer lateral margin gently convex; separated from first epibranchial tooth by V-shaped cleft; first epibranchial tooth distinct, wide, second epibranchial tooth just visible as low lobe (Fig. 2A, B); merus of third maxilliped subovate; exopod slender, flagellum elongate, longer than width of merus (Fig. 5A); outer surfaces of palm of chela covered with numerous small rounded granules (except at bases of pollex and dactylus), with cluster of closely packed larger ones on lower half, just posterior to pollex; inner surface with prominent flange-like transverse ridge lined by large rounded tubercles, surface of flange concave; fingers distinctly longer than palm, dorsal margin of dactylus with 15–17 small sharp granules arranged unevenly on surface, not in a row; dactylus with broad median molariform tooth on cutting edge; pollex with 2 distinct teeth on proximal half of cutting edge (Fig. 3A–D); ambulatory merus with sharp subdistal spine on dorsal margin, surface weakly rugose, propodus slender, relatively long; P4 merus length 3.0 times width; P2 propodus and dactylus without dense tuft of setae on flexor margins (Figs 2A, 3E, F); pleon broadly triangular; somite 3 widest, somite 6 wide, width to length ratio 2.6–2.7, lateral margins convex; telson triangular, 0.8–0.9 times longer than broad, lateral margins gently convex (Fig. 4B, C); G1 relatively slender, distal part bent at angle of ca. 50° along longitudinal axis, outer margin of subdistal part gently convex, without any shelf-like feature, distal chitinous elongate, tapering sharply in lateral view, subspatuliform in marginal view with distinct median cleft distally (Fig. 5B–I); vulvae positioned close to each other, opening laterally oblique (Fig. 4F).

##### Females and variation.

The denser row of setae lining the median postfrontal lobes is usually distinct in this species, with those on the lateral lobes usually shorter and sparser. The carapace of the females and subadult males do not differ substantially from the holotype and have more slender chelae (Fig. 4D). The female P2 also lacks tufts of setae on the flexor margins of the propodus and dactylus (Fig. 4D). The female pleon covers most of the thoracic sternum, and the semicircular telson is deeply inserted into the distal margin of somite 6 (Fig. 4E). The vulvae are large and positioned close to each other; with each gently swollen with smooth surface, and the opening laterally oblique in position close to each other, opening laterally oblique (Fig. 4F).

##### Colour.

**Figure 1.**
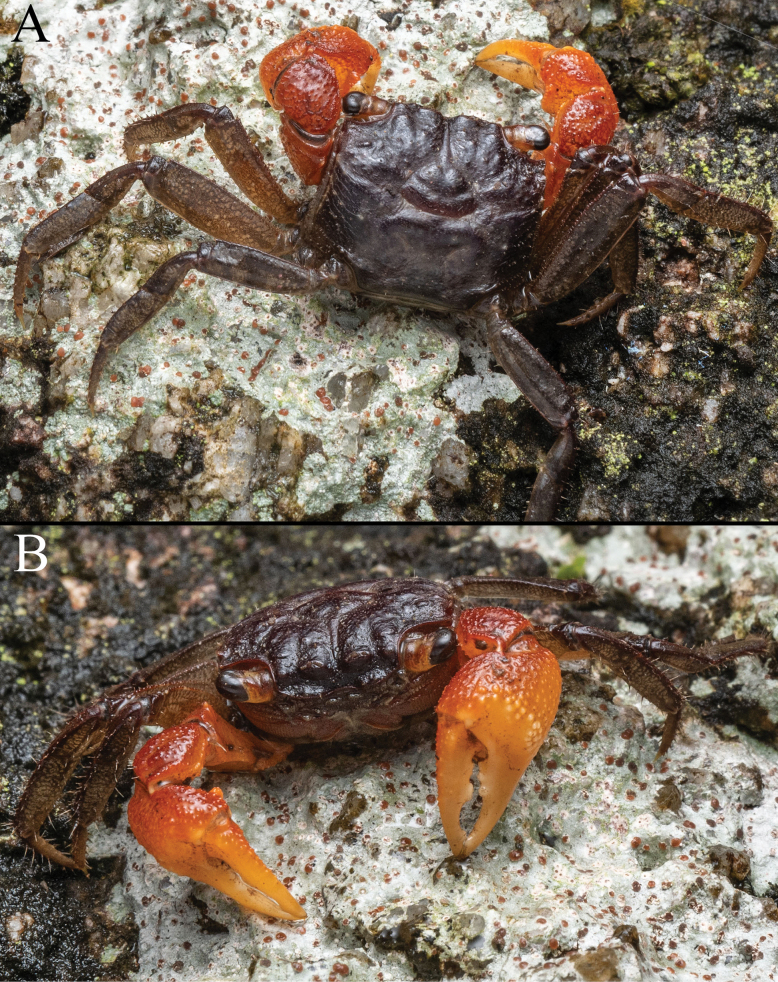
*Geosesarma wongi* sp. nov., holotype male (12.7 × 11.3 mm) (ZRC 2025.0023), Perak. Colour in life. Photograph: Francis Seow-Choen.

In life, the carapace and legs are dark brown, the cheliped is dark orange, and the fingers are a lighter orange. The ventral surfaces are light brown with the cornea black (Fig. 1).

**Figure 2.**
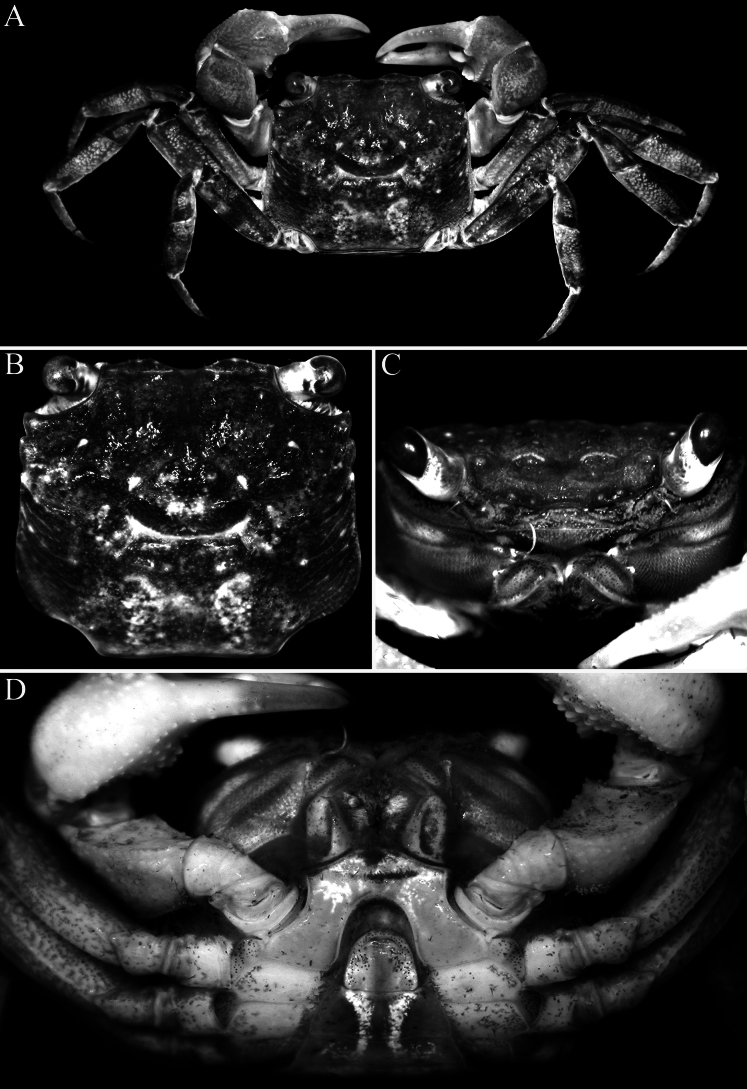
*Geosesarma wongi* sp. nov., holotype male (12.7 × 11.3 mm) (ZRC 2025.0023), Perak. **A.** Dorsal habitus; **B.** Dorsal view of carapace; **C.** Frontal view of cephalothorax; **D.** Ventral surface of cephalothorax.

**Figure 3.**
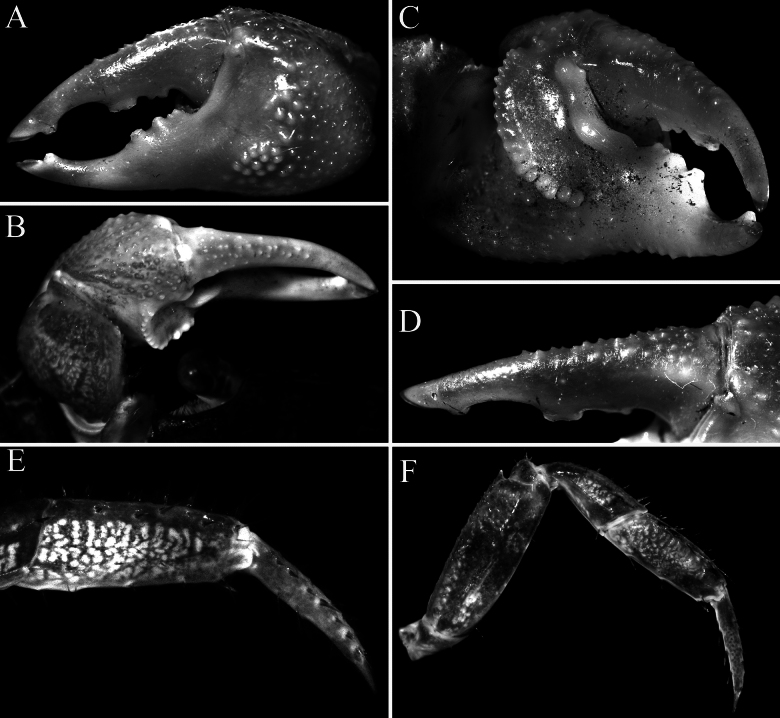
*Geosesarma wongi* sp. nov., holotype male (12.7 × 11.3 mm) (ZRC 2025.0023), Perak. **A.** Outer view of left chela; **B.** Dorsal view of left cheliped; **C.** Inner view of left chela showing crest; **D.** Dactylus of left chela (subdorsal view); **E.** Propodus and dactylus of right P2; **F.** Right P5.

**Figure 4.**
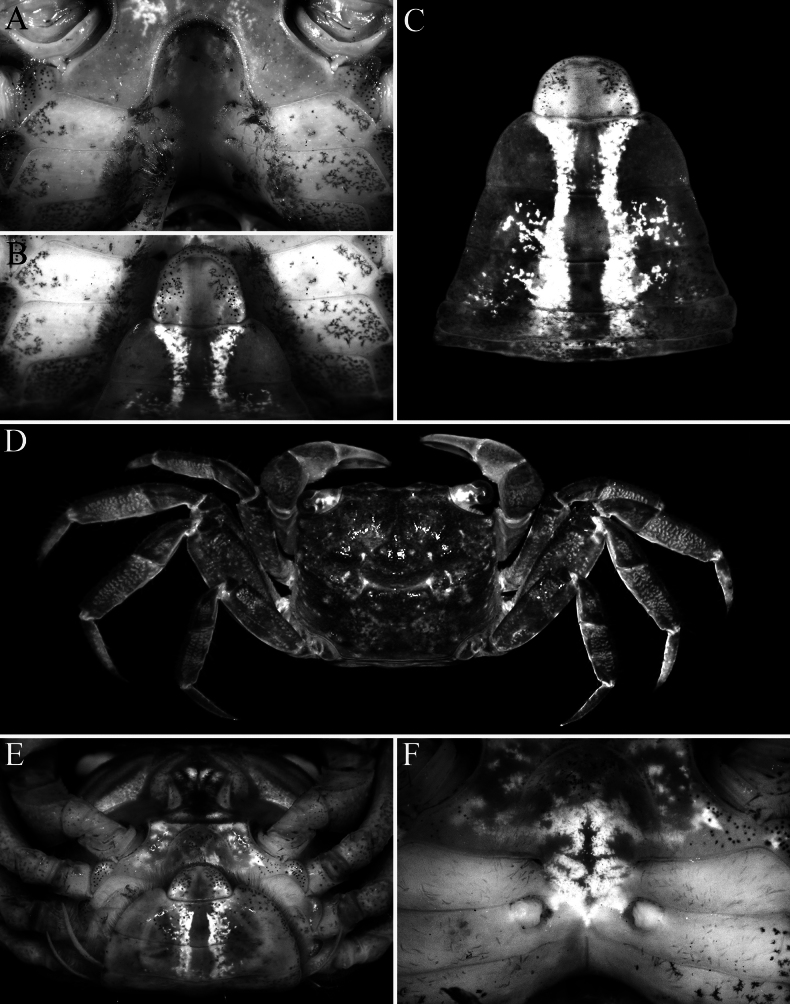
*Geosesarma wongi* sp. nov. **A–C.** Holotype male (12.7 × 11.3 mm) (ZRC 2025.0023), Perak; **D–F.** Paratype female (12.6 × 10.8 mm) (ZRC 2025.0027), Perak; **A.** Male sternopleonal cavity; **B.** Male sternopleonal cavity and pleonal somites 5, 6 and telson; **C.** Male pleonal somites 2–6 and telson (telson slightly bent anteriorly); **D.** Female dorsal habitus; **E.** Ventral surface of female cephalothorax showing pleon; **F.** Female sternopleonal cavity showing vulvae.

**Figure 5.**
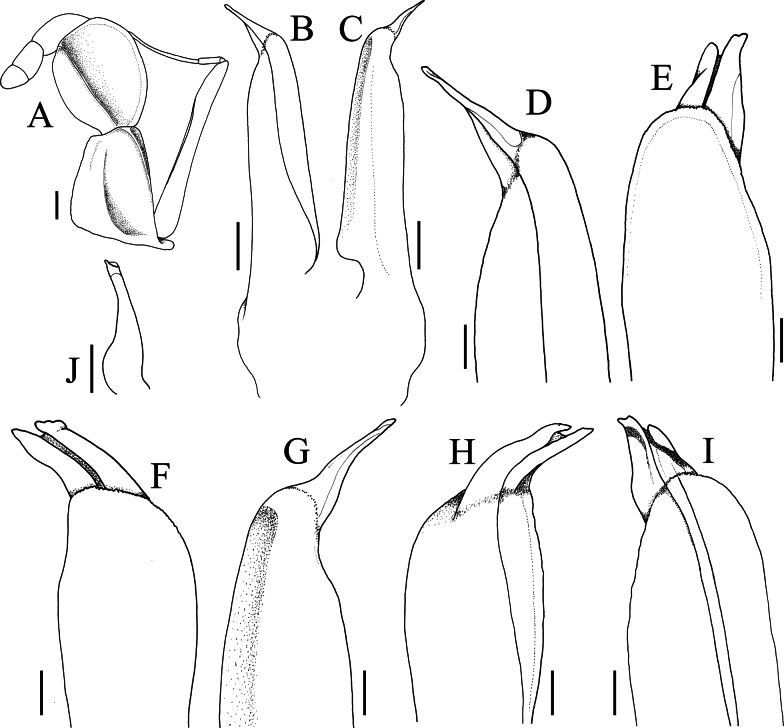
*Geosesarma wongi* sp. nov., holotype male (12.7 × 11.3 mm) (ZRC 2025.0023), Perak. **A.** Left third maxilliped; **B.** Left G1 (dorsal view); **C.** Left G1 (ventral view); **D.** Distal part of left G1 (dorsal view); **E, F.** Distal part of left G1 (mesial views); **G.** Distal part of left G1 (ventral view); **H, I.** Distal part of left G1 (outer lateral views); **J.** Left G2 (dorsal view). All structures denuded. Scale bars: 0.5 mm (**A–C, J**); 0.2 mm (**D–I**).

##### Remarks.

In the subrectangular shape and armature on the carapace, with low rounded granules and tubercles, and the presence of a well-developed transverse crista on the inner surface of the male chela, *G. wongi* sp. nov. belongs to the same group of Southeast Asian species as *G. amphinome* (De Man, 1899) [West Kalimantan], *G. peraccae* (Nobili, 1903) [Singapore and Johor, Peninsular Malaysia], *G. sarawakense* (Serène, 1968) [Sarawak], *G. cataracta* Ng, 1986 [Perak, Malaysia] and *G. pylaemenes* Ng, 2015 [West Kalimantan].

In *G. wongi* sp. nov., the transverse crista on the inner surface of the male chela is very prominent, especially in the holotype, being much stronger and more produced than in the other species (Fig. 3B, C) (cf. Fig. 9A, D, E; Ng 2015: figs 2F, 3D, F, 5F). The size of the transverse crista on the inner surface of the male chela, however, varies with the size of the specimen. It is most prominent in the largest holotype male (12.7 × 11.3 mm, ZRC 2025.0023; Fig. 3B, C) but is distinctly smaller and less well-developed in smaller males measuring 10.1 × 8.9–10.6 × 9.5 mm (ZRC 2025.0027). In these smaller males, the crista is more similar to those of the other species discussed earlier possessing this character; and as such, the differences observed may be due to the maturity of the males.

**Figure 6.**
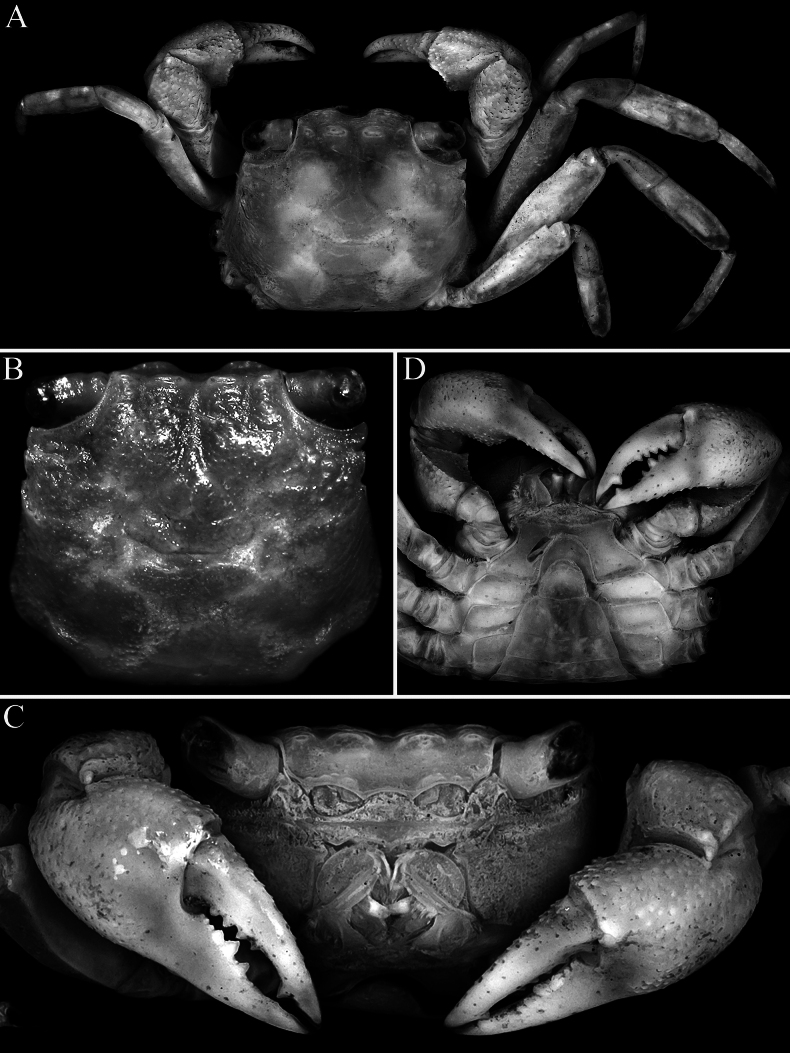
*Geosesarma peraccae* (Nobili, 1903), lectotype male (11.8 × 9.8 mm) (MRSN Cru 1421a), Singapore. **A.** Dorsal habitus; **B.** Dorsal view of carapace; **C.** Frontal view of cephalothorax and chelipeds; **D.** Ventral surface of cephalothorax. Photographs: **A, D, C** by Giuliano Giacobelli & Marco Bernardi.

**Figure 7.**
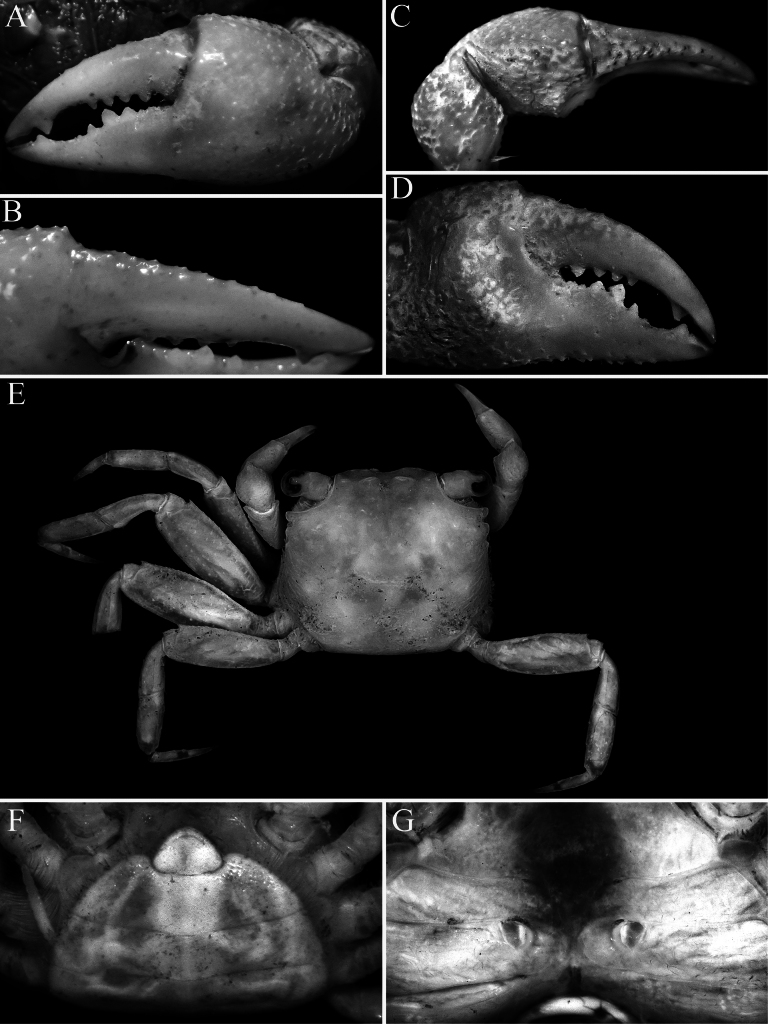
*Geosesarma peraccae* (Nobili, 1903). **A–D.** Lectotype male (11.8 × 9.8 mm) (MRSN Cru 1421a), Singapore; **E.** Paralectotype female (9.1 × 7.9 mm) (MRSN Cru 1421b), Singapore; **F, G.** Female (12.6 × 10.7 mm) (ZRC 1990.0518), Singapore; **A.** Outer view of left chela; **B.** Dactylus of left chela (subdorsal view); **C.** Dorsal view of left cheliped; **D.** Inner view of left chela showing crest; **F.** Ventral surface of female cephalothorax showing pleon; **G.** Female sternopleonal cavity showing vulvae. Photograph: (**E**) by Giuliano Giacobelli & Marco Bernardi.

**Figure 8.**
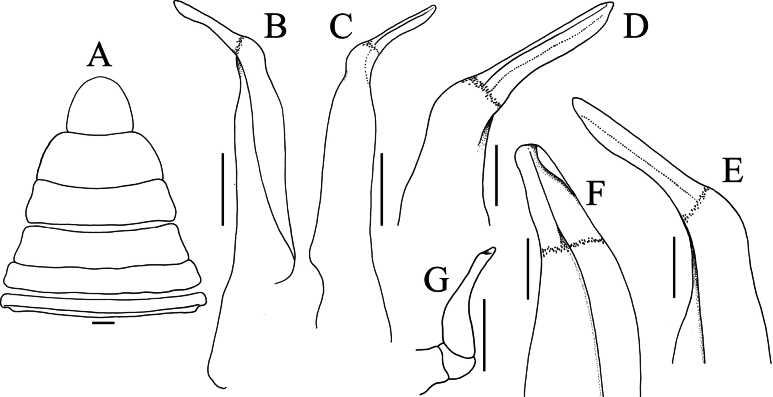
*Geosesarma peraccae* (Nobili, 1903), lectotype male (11.8 × 9.8 mm) (MRSN Cru 1421a), Singapore. **A.** Male pleon; **B.** Left G1 (dorsal view); **C.** Left G1 (ventral view); **D.** Distal part of left G1 (dorsal view); **E.** Distal part of left G1 (ventral view); **F.** Distal part of left G1 (mesial view); **G.** Left G2 (dorsal view). All structures are denuded. Scale bars: 0.5 mm (**A–C, G**); 0.2 mm (**D–F**).

**Figure 9.**
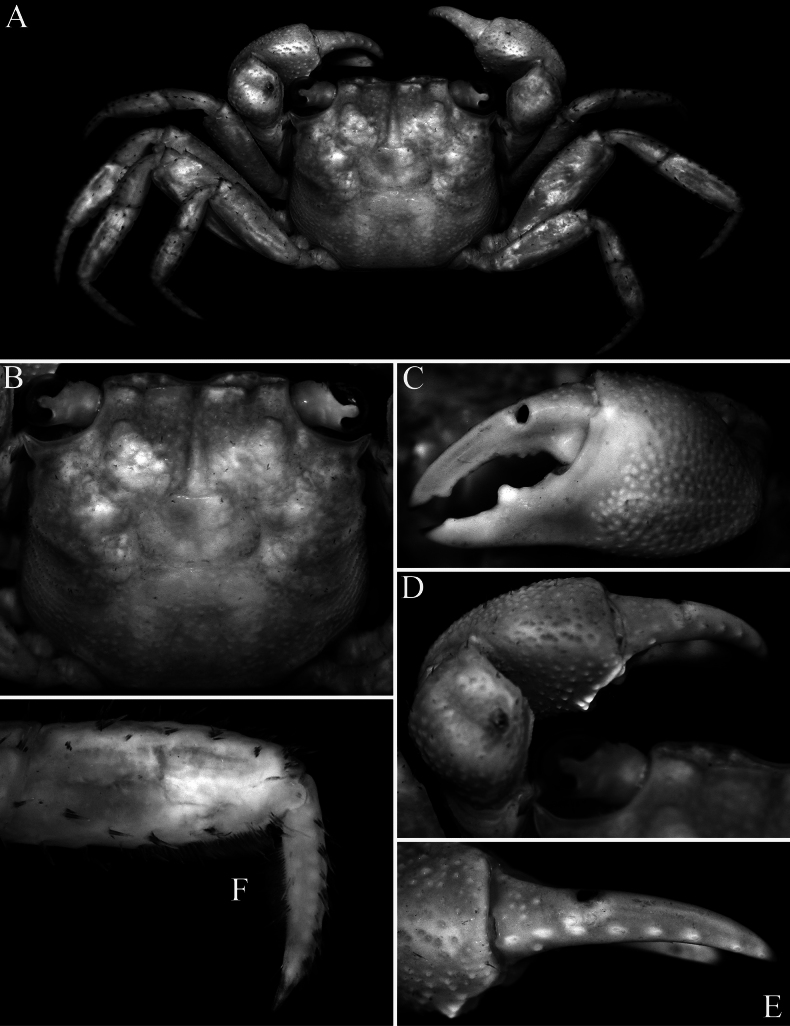
*Geosesarma cataracta* Ng, 1986, holotype male (12.4 × 10.9 mm) (ZRC 1985.1760), Perak. **A.** Dorsal habitus; **B.** Dorsal view of carapace; **C.** Outer view of left chela; **D.** Dorsal view of left cheliped; **E.** Dactylus of left chela (dorsal view); **F.** Propodus and dactylus of right P2.

The other species in this group also have relatively lower urogastric regions and are not as raised (Fig. 9A, B; Ng 2015: figs 1A, B, 3A, B, 5A, B, 6A, B) as in *G. wongi* sp. nov. (Figs 1A, 2B). The armature of the cutting edges of the fingers of the adult chela is also different in *G. peraccae* and *G. wongi* sp. nov. In *G. peraccae*, there is no median molariform tooth on the dactylar finger, and there are only uneven teeth on the proximal part of the pollex (Figs 6C, D, 7A, D; Ng 1988: fig. 56B; Ng 2015: fig. 5E, F). In *G. wongi* sp. nov., on the other hand, the median molariform tooth is distinct, and there are two smaller molariform teeth on the proximal part of the pollex (Fig. 3A). The male pleon of *G. wongi* sp. nov. is proportionately much wider compared to most of the species in this group, especially somite 6 (Fig. 4B, C) (cf. Ng 2015: figs 1E, 2C, 4B, 7B). Only in *G. peraccae* does the male pleon share similar proportions (cf. Fig. 8A; Ng 2015: fig. 5D). The G1 of *G. wongi* sp. nov. has the distal chitinous part spatuliform, and this character is also shared by the species with a well-developed transverse crista on the inner surface of the male chela. In *G. wongi* sp. nov., the distal chitinous part is distinctly flatter and in lateral view, the structure appears narrow and acutely tapered to the tip (Fig. 5B–D, G). In most other species with similar G1s, the distal chitinous part is more curved along the longitudinal axis, and in lateral view the structure is prominently wider (cf. Ng 2015: figs 2G–K, 4D–G, 7D–F). The G1 distal chitinous part of *G. peraccae* is most similar in form to that of *G. wongi* sp. nov., being narrow in lateral view (Fig. 8B–E; Ng 1988: fig. 56D, F) but in the dorso-mesial view, the structure is distinctly narrower, and the tip is entire (Fig. 8B–F; Ng 1988: fig. 56E). In *G. wongi* sp. nov., the G1 distal chitinous part is very wide in the dorso-mesial view and there is a prominent median cleft at the tip (Fig. 5E, F, H, I). In addition, the subdistal part of the G1 is proportionately stouter in *G. wongi* sp. nov. (Fig. 5B, C) than in *G. peraccae* (Fig. 8B, C; Ng 1988: fig. 56D, F).

**Figure 10.**
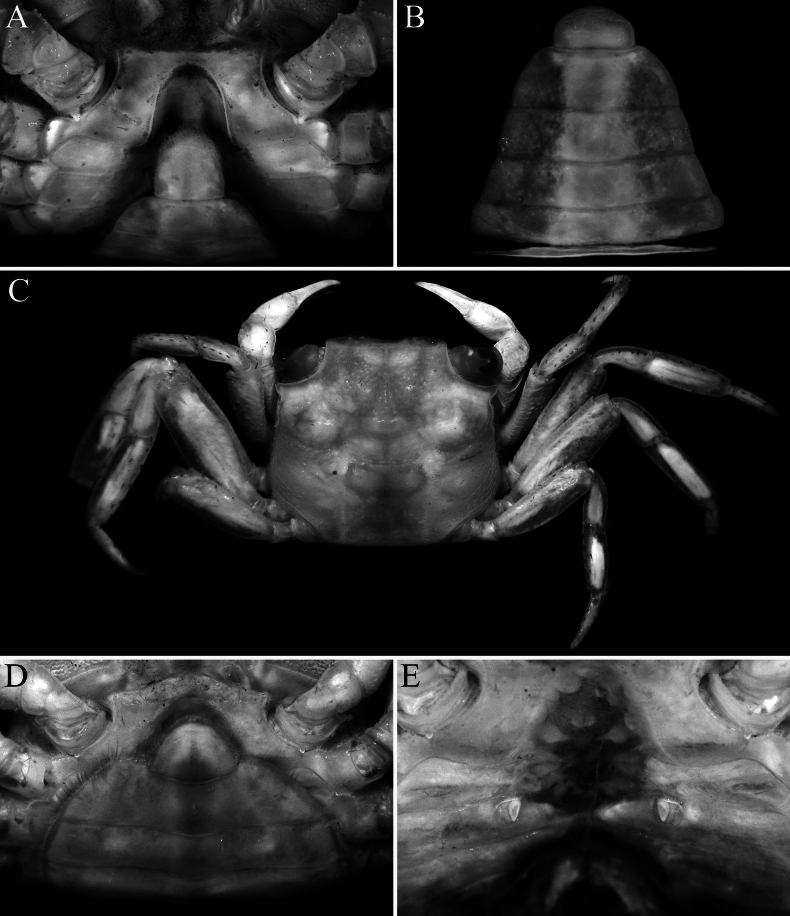
*Geosesarma cataracta* Ng, 1986. **A, B.** Holotype male (12.4 × 10.9 mm) (ZRC 1985.1760), Perak; **C–E.** Paratype female (10.5 × 9.5 mm) (ZRC 1985.1762), Perak; **A.** Sternopleonal cavity and pleonal somites 5, 6 and telson; **B.** Pleonal somites 1–6 and telson (telson bent anteriorly); **C.** Female dorsal habitus; **D.** Ventral surface of female cephalothorax showing pleon; **E.** Female sternopleonal cavity showing vulvae.

The only member of the species-group known in the state of Perak is *G. cataracta* from Bukit Larut (= Maxwell Hill) which is about 90 km northwest of the type locality of *G. wongi* sp. nov. *Geosesarma wongi* sp. nov. can easily be distinguished by the external orbital tooth being broadly triangular, with the tip not extending beyond the lateral margin and directed anteriorly (Fig. 2A, B) (versus external orbital tooth more acutely triangular in form, with the tip extending beyond the lateral margin and directed obliquely in *G. cataracta*; Ng 1988: fig. 50A); the protogastric region is only gently convex and not raised (Figs 1A, 2B) (versus region raised by presence of large flattened tubercles in *G. cataracta*; Fig. 9A, B); the outer face of the male palm has a distinct cluster of closely packed rounded granules on the lower half, posterior to the base of pollex, the dactylus has large molariform median tooth on the cutting edge, the dorsal margin of the dactylus has 15–17 small sharp granules arranged unevenly on surface and the pollex has two submolariform teeth on the proximal half of the cutting edge (Fig. 3A–D) (versus outer surface of palm with uniformly distributed granules and no obvious cluster of closely packed granules, there is no molariform tooth on the cutting edge of the dactylus, the dorsal margin of the dactylus has 7 or 8 low tubercles arranged in a longitudinal row and the pollex has one rounded median tooth on the cutting edge in *G. cataracta*; Ng 1988: fig. 59B); the flexor margins of the propodus and dactylus of the adult male P2 do not possess a fringe of stiff setae (Fig. 3E) (versus with dense fringe of stiff setae in *G. cataracta*; Ng 1988: fig. 59G); the male telson is proportionately shorter (Figs 2D, 4B) (versus telson more elongate in *G. cataracta*; Fig. 10A); the G1 has the chitinous distal part more elongate and spatuliform (Fig. 5B–D, G, H) (versus distal part shorter and acutely tapering in *G. cataracta*; Ng 1988: fig. 59D–F); and the vulvae are relatively larger and positioned closer to each other (Fig. 4F) (versus distinctly smaller and positioned further apart in *G. cataracta*; Fig. 10E).

The type material of *G. wongi* sp. nov. was collected with numerous specimens of *G. malayanum*, indicating that both species are sympatric, although their exact habitats are unknown.

**Figure 11.**
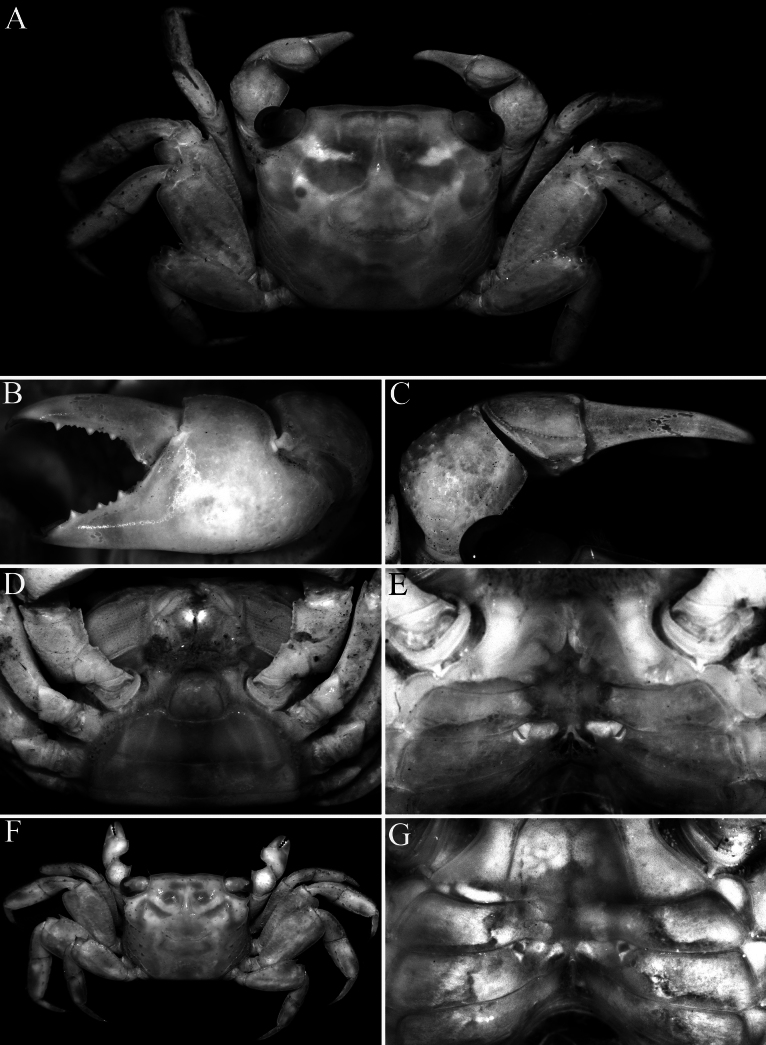
**A–E.***Bresedium* sp., female (12.3 × 10.8 mm) (ZRC 1985.1761) [paratype of *Geosesarma cataracta* Ng, 1986] **F.***Bresedium laevimanum* (Zehntner, 1894), female (12.2 × 10.7 mm) (ZRC 1972.3.7.30), Kuching, Sarawak, Malaysia, coll. M.W.F. Tweedie, January 1949; **A, F.** Dorsal habitus; **B.** Outer view of left chela; **C.** Dorsal view of left cheliped; **D.** Ventral surface of cephalothorax showing pleon; **E, G.** Sternopleonal cavity showing vulvae.

The type series of *G. cataracta* was re-examined, and it was determined that the largest female in the paratype series, measuring 12.3 × 10.8 mm (ZRC 1985.1761), is actually not a species of *Geosesarma*. It has a very smooth carapace, an almost straight front with two postorbital lobes (rather than four), broad ambulatory meri as well as more setose ambulatory propodi and dactyli (Fig. 11A), and the vulva has a distinct structure, with two closely placed vulvar covers with a slit like opening for the opening (Fig. 11E).

The vulva of ZRC 1985.1761, with the median slit between the two vulvar covers, is most similar to that reported for *Parasesarma bengalense* (Davie, 2003) from the eastern Indian Ocean by Shahdadi and Schubart (2017: fig. 10C), although the slit for this species is slightly wider. While the carapace shape of ZRC 1985.1761 agrees with that of *P. bengalense*, the latter species has a much rougher and more setose carapace and the outer surface of the chela is prominently granulose, even in females (cf. Davie 2003: figs 1C, 2A, C) and the female telson is strongly sunk into the distal margin of somite 6 (Shahdadi and Schubart 2017: fig. 9C). In ZRC 1985.1761, the outer surface of the chela is smooth and female telson is only partially inserted into the distal margin of somite 6 (Fig. 11D). The carapace of ZRC 1985.1761 also resembles that of *Fasciarma fasciatum* (Lanchester, 1900) [Southeast Asia], a species common in back mangroves, but the ambulatory legs of this species is prominently longer and more slender (Shahdadi and Schubart 2017: fig. 15A, B, G) and the vulva is mushroom-shaped, without a median slit (Shahdadi and Schubart 2017: fig. 10F).

The carapace and ambulatory legs of ZRC 1985.1761 closely resembles species of *Pseudosesarma* Serène & Soh, 1970 s. str. (cf. Schubart and Ng 2020). The relatively smooth carapace surfaces and the presence of an epibranchial tooth are associated with species like *P. crassimanum* (De Man, 1887) [Southeast Asia] and *P. glabrum* Ng, Rani & Nandan, 2017 [eastern India]. *Pseudosesarma crassimanum* has a carapace which is somewhat more setose, the surfaces of the ambulatory legs are more rugose, the inner angle of the carpus of the cheliped has a sharp tooth (Schubart and Ng 2020: fig. 22D). The very smooth carapace and blunt inner angle of the carpus of the cheliped of ZRC 1985.1761 is closer in form to the condition in *P. glabrum* but the ambulatory merus in this species is more rugose (cf. Schubart and Ng 2020: fig. 22F). The vulvae of these *Pseudosesarma* species are also different in structure, with the gap between the vulvar covers much wider (Schubart and Ng 2020: fig. 44B, C). In any case, all known *Pseudosesarma* species have the outer surfaces of their chelae distinctly granular in both sexes (cf. e.g., Schubart and Ng 2020: fig. 27D, F), and as such, ZRC 1985.1761 is unlikely to be a member of the genus.

The smooth carapace and the smooth unarmed outer surface of the chela of ZRC 1985.1761 are very similar to those in *Bresedium laevimanum* (Zehntner, 1894) (= *Sesarma sediliensis* Tweedie, 1940) as rediagnosed by Schubart and Ng (2020: figs 24E–H, 29D–F). It cannot be *B. laevimanum* as the inner angle of the carpus of the cheliped of this species has a sharp tooth (Fig. 11F; Schubart and Ng 2020: fig. 24E–H) (blunt in the present specimen, Fig. 11A, C) and the vulva is formed by two triangular vulvar cover plates with a wide gap between them (Fig. 11G) (a narrow slit between the covers in the present specimen, Fig. 11E). Specimen ZRC 1985.1761 is thus not *Bresedium laevimanum* but probably an allied taxon. The taxonomy of *Bresedium*, which now contains three species (see Li et al. 2020; Shahdadi and Ng 2025), is now being revised by H.-T. Shih and colleagues, and specimen ZRC 1985.1761 is provisionally placed there. *Bresedium* species like *B. laevimanum* are known from back mangroves and can be found some distance from the shore, but its presence in a waterfall on Bukit Larut is noteworthy as this location is about 15 km from the nearest mangroves.

## Supplementary Material

XML Treatment for Geosesarma

XML Treatment for Geosesarma wongi

## ﻿Additional information

### Use of AI

No use of AI was reported.

### Author ORCIDs

Peter K. L. Ng https://orcid.org/0000-0001-5946-0608

### Data availability

All of the data that support the findings of this study are available in the main text.
